# Concentrations and bioconcentration factors of leaf microelements in response to environmental gradients in drylands of China

**DOI:** 10.3389/fpls.2023.1143442

**Published:** 2023-03-02

**Authors:** Yahui Zhang, Shubin Xie, Xiaoting Wang, Muhammad Adnan Akram, Weigang Hu, Longwei Dong, Ying Sun, Hailing Li, Abraham Allan Degen, Junlan Xiong, Jinzhi Ran, Jianming Deng

**Affiliations:** ^1^ State Key Laboratory of Herbage Improvement and Grassland Agro-ecosystems (SKLHIGA), College of Ecology, Lanzhou University, Lanzhou, China; ^2^ School of Economics, Lanzhou University, Lanzhou, China; ^3^ Desert Animal Adaptations and Husbandry, Wyler Department of Dryland Agriculture, Blaustein Institutes for Desert Research, Ben-Gurion University of Negev, Beer Sheva, Israel

**Keywords:** Northern China, aridity, leaf microelements, bioconcentration factor, indicator plant species

## Abstract

Determining response patterns of plant leaf elements to environmental variables would be beneficial in understanding plant adaptive strategies and in predicting ecosystem biogeochemistry processes. Despite the vital role of microelements in life chemistry and ecosystem functioning, little is known about how plant microelement concentrations, especially their bioconcentration factors (BCFs, the ratio of plant to soil concentration of elements), respond to large-scale environmental gradients, such as aridity, soil properties and anthropogenic activities, in drylands. The aim of the present study was to fill this important gap. We determined leaf microelement BCFs by measuring the concentrations of Mn, Fe, Ni, Cu and Zn in soils from 33 sites and leaves of 111 plants from 67 species across the drylands of China. Leaf microelement concentrations were maintained within normal ranges to satisfy the basic requirements of plants, even in nutrient-poor soil. Aridity, soil organic carbon (SOC) and electrical conductivity (EC) had positive effects, while soil pH had a negative effect on leaf microelement concentrations. Except for Fe, aridity affected leaf microelement BCFs negatively and indirectly by increasing soil pH and SOC. Anthropogenic activities and soil clay contents had relatively weak impacts on both leaf microelement concentrations and BCFs. Moreover, leaf microelement concentrations and BCFs shifted with thresholds at 0.89 for aridity and 7.9 and 8.9 for soil pH. Woody plants were positive indicator species and herbaceous plants were mainly negative indicator species of leaf microelement concentrations and BCFs for aridity and soil pH. Our results suggest that increased aridity limits the absorption of microelements by plant leaves and enhances leaf microelement concentrations. The identification of indicator species for the response of plant microelements to aridity and key soil characteristics revealed that woody species in drylands were more tolerant to environmental changes than herbaceous species.

## 1 Introduction

All 14 essential elements including 6 macroelements and 8 microelements, are important for plant ontogenetic processes such as growth, maintenance and reproduction (Marschner, 2012; Luo et al., 2016). Although there are numerous studies on the variation in concentrations of both plant and soil macroelements and on their impacts on plant growth and functions (Delgado-Baquerizo et al., 2013; Jiao et al., 2016; Li et al., 2022), little is known on how the concentrations of plant and soil microelements respond concomitantly to an environmental gradient.

Microelements play vital roles in plant ontogenetic growth and functions by influencing enzyme activities of biochemical reactions in organisms (Huang et al., 2020). For example, more than 35 enzymes, which are involved in different physiological functions and redox reactions, are activated by manganese (Mn) (Kabata-Pendias and Szteke, 2015). As a redox active metal, iron (Fe) is essential for many biological processes, including photosynthesis, mitochondrial respiration, nitrogen assimilation, and hormone biosynthesis (Marschner, 2012). Nickel (Ni) is needed for many prokaryotic enzymes and is involved in nitrogen (N) metabolism, while copper (Cu) is indispensable for photosynthesis, carbon (C) and N metabolism, oxidative stress protection and cell wall synthesis (Hänsch and Mendel, 2009). Zinc (Zn) is an essential component of enzymes and is involved in protein synthesis, energy production, and maintenance of the structural integrity of bio-membranes (Marschner, 2012). Ni, Cu and Zn are heavy metals in the soil (Qin et al., 2021), and either excessive or deficient contents can constrain plant growth, and physiological and metabolic processes (Mohamed et al., 2003; Sardar et al., 2013).

Recent studies have focused on the bioconcentration factor (BCF) of elements in plants to determine the capacity of plants to absorb metallic elements (Lin et al., 2016; Bonanno et al., 2017; Thanh-Nho et al., 2019; Zhang et al., 2019; Akram et al., 2021). The capacity of plants to absorb metallic elements is dependent not only on the plants, but also on soil temperature, texture, cation exchange capacity, organic matter, and the concentrations of available elements (Gupta et al., 2019). Furthermore, aridity can also affect the soil available elements in drylands indirectly through its effects on soil pH, organic matter and clay content (Moreno-Jiménez et al., 2019). Increasing aridity due to climate change, therefore, can have a strong influence on global biogeochemical cycles and ecosystems (Jiao et al., 2016), but its effect on the concentration and BCF of metallic elements in plants remains unclear. Additionally, anthropogenic activities related to agriculture and industry, and climate change are major drivers of global environmental change (Delgado-Baquerizo et al., 2016), which further modifies nutrient element cycling in an ecosystem. However, little is yet known on the effects of aridity and anthropogenic activities on the absorption of metallic elements by plants. It was reported that anthropogenic activities can have a positive impact on the atmospheric deposition of potassium (K), while aridity can have a negative effect on K absorption by plants in drylands (Sardans and Peñuelas, 2015).

Aridity has increased in the past decades in many drylands (Huang et al., 2016), which is of concern, as drylands are fragile and sensitive to climate change and human activities (Hu et al., 2021). Previous studies examined the effects of water, salinity and temperature stress on plants in drylands (Perri et al., 2018; Peguero-Pina et al., 2020). However, metallic elements, including microelements, in soil are usually very rich to cause stress on plant ontogenetic growth and function in drylands because of the serious soil salinization. Therefore, an understanding of microelement cycling would be beneficial in maintaining and improving the plant community structure and ecosystem functions of the terrestrial biosphere under global change (Cai et al., 2017).

Site-specific environmental factors can influence concentrations and thresholds of biological tolerance of plant nutrient elements (Ren et al., 2019). Determining the variations in leaf microelement concentrations and BCFs along environmental gradients enables the calculations of ecological thresholds, and, thereby, to understand abrupt shifts in ecological responses of microelements (Groffman et al., 2006). Generally, there is a shift in dominant plant species from herbaceous to desert shrub species with increasing aridity in dryland ecosystems (Yao et al., 2021b). Herbaceous plant species, with faster growth rates, typically grow in nutrient-rich environments, and display less tolerance to environmental stresses than woody plant species (Rebele, 2013). Woody species, with slower growth rates, tend to grow in nutrient-poor environments, and display a higher tolerance to environmental stresses than herbaceous species (Sun et al., 2020). Consequently, in a harsh environment, Xiong et al. (2021) found that species with weak stress tolerance can serve as negative indicators, while stress-tolerant species can serve as positive indicators of leaf C, N and P concentrations along an increasing environmental stress gradient. Nevertheless, it remains unclear whether leaf microelement concentrations follow the same pattern in drylands.

We selected 33 sites and 111 plant samples from 67 species across the drylands of northern China to determine the response of plant leaf microelement (Mn, Fe, Ni, Cu and Zn) concentrations and BCFs to environmental gradients. Based on previous studies, we hypothesized that: (1) leaf microelement concentrations are maintained within relatively normal ranges to satisfy the basic requirements of plants to survive and reproduce; (2) leaf microelement concentrations and BCFs are mediated by aridity, human impact index (HII), soil physical properties and soil chemical contents; (3) environmental gradients cause abrupt shifts in the response of plant element concentrations to the ecological environment; that is, there are thresholds for plant leaf microelement concentrations and BCFs along major environmental gradients; and (4) as adaptation strategies to environmental stress differ among plant taxa (Xiong et al., 2021), woody species could serve as positive indicators and herbaceous species as negative indicators for leaf microelement concentrations and BCFs along aridity and soil pH gradients; whereas, herbaceous species could serve as positive indicators and woody species as negative indicators along SOC gradients.

## 2 Materials and methods

### 2.1 Site description, collection of samples

**Figure 1:**
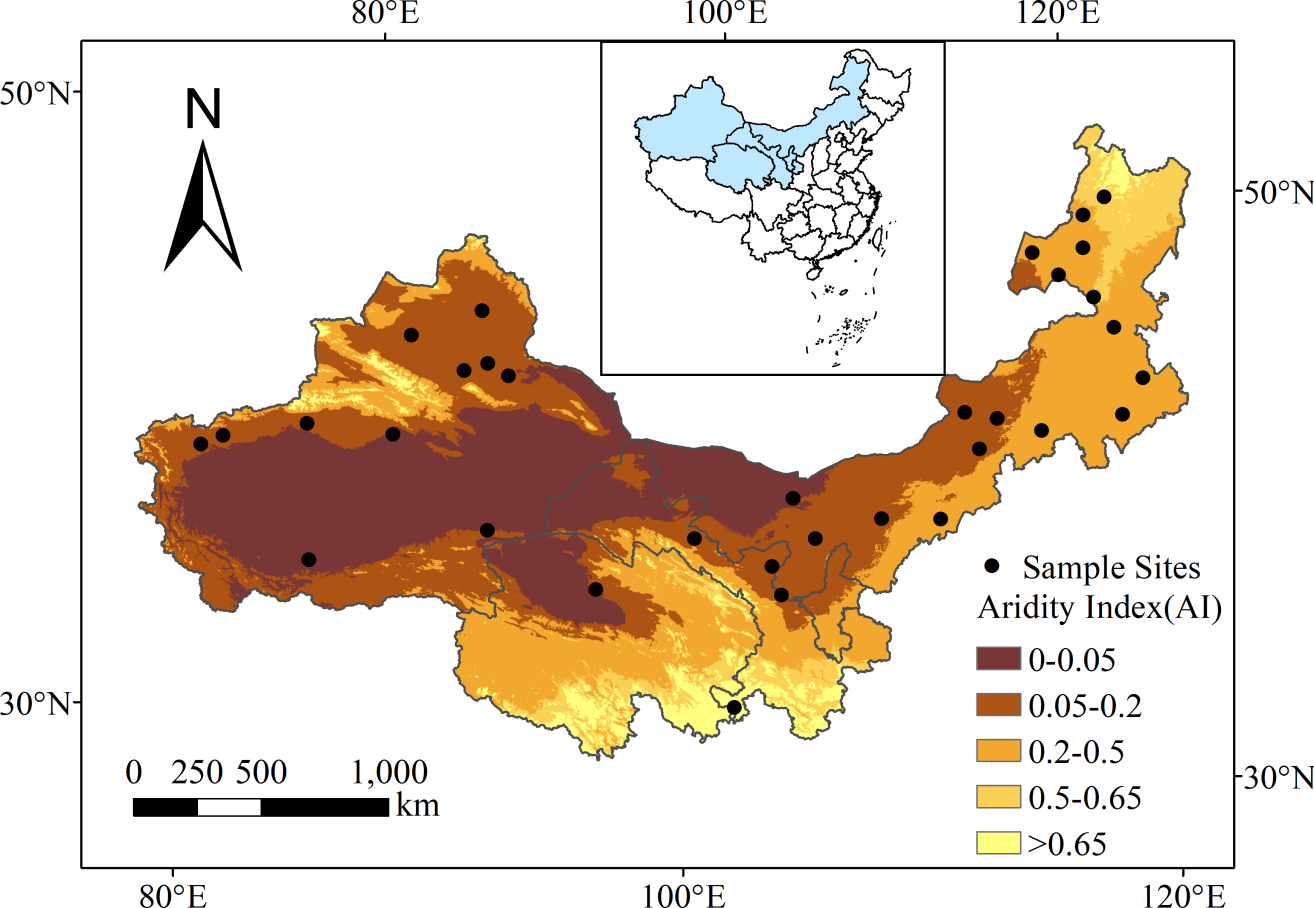
Distribution of the 33 sampling sites in drylands of northern China. The background map is aridity index (AI; generated by ArcGIS 10.3). Inset map shows China with the study area in blue.

Thirty-three sites were selected across Xinjiang, Inner Mongolia, Gansu, and Qinghai provinces in northern China (**Figure 1**). The region extends between 33.67°N to 50.70°N and 76.38°E to 121.68°E, with elevations ranging between 194 and 3567 m above sea level (**Figure 1**). Mean annual precipitation ranges between 35 and 474 mm, mean annual temperature ranges between -3.8 and 12.1 °C, and aridity [1 – aridity index, where aridity index = precipitation/potential evapotranspiration (Delgado-Baquerizo et al., 2018)] ranges between 0.28 and 0.98. The main vegetation types in the sample sites included desert, desert grassland, typical grassland and alpine grassland. There were 14 types of soil, including cambisols, chemozems and calcisols.

Leaves and soils were sampled during the growing seasons (June to September) from 2013 to 2017, using standard protocols described by Deng et al. (2006) and Chen et al. (2019). At least three representative and relatively homogeneous vegetation quadrats, each 30 m × 30 m, were selected randomly at each site. Dominant plant species were identified in each quadrat, and at least 5 mature individuals of each species were collected. In total, there were 67 species, belonging to 57 genera and 25 families. Five core soil samples at a depth of 0-20 cm were collected randomly in non-vegetated areas (bare land) and under the canopy of dominant plant species in each quadrat. The five soil samples of each type were combined.

### 2.2 Data sources and chemical analyses

An *in-situ* portable global positioning system recorded the coordinates of each site. Aridity data were extracted from the WorldClim 2.0 database (https://www.worldclim.org). Human influence index (HII), ranging from 0 to 100 (0 is the minimum and 100 the maximum HII value) was obtained from Last of the Wild (v2) (Wildlife Conservation Society (WCS) and Center for International Earth Science Information Network (CIESIN) at Columbia University, 2005). Soil clay content was acquired from WISE30sec database (Batjes, 2016).

Soil samples were air-dried, and stones, roots, leaves and debris were removed. After homogenization, the soil was sieved through a 2 mm nylon mesh for measurements of soil variables. For the determination of soil Mn, Fe, Ni, Cu and Zn contents, a portion of the soil was ground in an agate mortar, sieved through a 0.074-mm nylon mesh, and stored in a desiccator. Intact leaves were collected from the dominant plant species, rinsed three times with double-distilled water to remove dust and soil, oven-dried at 60 °C for 72 h to constant weight, ground to a fine powder, and kept in a desiccator before measurements.

Soil pH and soil electrical conductivity (EC) were determined by the soil: distilled water method using a ratio of 1:2.5 and 1:5, with a pH meter (Sartorius PB-10, Göttingen, Germany) and EC meter (DDSJ-318, Yantai Stark Instrument, Yantai, China), respectively. Soil organic carbon (SOC) concentration was determined by the wet oxidation method: 0.5 g soil was digested with 5 mL K_2_Cr_2_O_7_ and 5 mL concentrated H_2_SO_4_ at 180 °C for 30 min, and then titrated with 0.5 M FeSO_4_ till endpoint.

To determine concentrations of Mn, Fe, Ni, Cu and Zn in soil and plant samples, different digestion procedures were used. For soil samples, an ultrapure mixture of HNO_3_ (6 mL) + HF (2 mL) + HCl (2 mL) was used, and for plant samples, an ultrapure mixture of HNO_3_ (6 mL) + H_2_O_2_ (2 mL) was used. Each sample (0.0500 g) was solubilized in a 50 mL Teflon tube, sealed, placed in a high efficiency anti-corrosive tube sheath with polytetrafluoroethylene (PTFE) coating, and digested at 192 °C for 1.5 h. The solution was evaporated to dryness, driven away by HNO_3_ acid, diluted with Milli-Q water to 50 g, and stored at 4 °C for further analysis. The concentrations of elements in the soil and plant samples were determined by inductively coupled plasma mass spectrometry (VG PQ ExCell; Thermo Elemental). The concentrations of the microelements are expressed as molar mass per unit dry mass (mol/kg).

Bioconcentration factor (BCF), the capacity of the plant to accumulate microelements in the leaf from soil (Pavel et al., 2014; Yang et al., 2015), was calculated as follows:


$$\text{Bioconcentration Factor (BCF)}=C_{leaf}/C_{soil}$$

where *C_leaf_* and *C_soil_* represent the concentrations of the same microelement in the leaf and soil, respectively.

### 2.3 Detection of phylogenetic signals of traits

To determine the influence of phylogeny on the accumulation of microelements by dryland plants, *K*-statistics were used to detect the phylogenetic signals (*K*-values) in relation to the leaf microelement concentrations and BCFs. *K*-statistics are based on the ‘Brownian motion’ model to estimate the relationship between the total variation and the random expectation of the variance of the traits (Blomberg et al., 2003). When *K* > 1, the trait is controlled mainly by phylogeny, as the phylogenetic signal of the trait is stronger than the Brownian motion model; however, when *K* < 1, the trait is influenced mainly by environmental factors. When using phylogenetic signals to measure functional traits, species in phylogenetic trees are usually arranged randomly 999 times, and a *K-*value is calculated each time. If the observed value is greater than the *K*-value of the null model (α < 0.05), then the phylogenetic signal of the trait is significant; however, if the observed value is lesser than the *K*-value of the null model (α > 0.05), then the phylogenetic signal is weak (Blomberg et al., 2003). The concentrations and BCFs of microelements were treated as continuous traits. Species mean values were used and analyses used ‘ape’ (Paradis et al., 2004) and ‘picante’ (Kembel et al., 2010) packages in R.

### 2.4 Data analyses and statistics

The data were tested for normality and homoscedasticity, and, where necessary, were log_10_-transformed before analysis. Subsequently, we used a variance inflation factor (VIF) to test for multi-collinearity, and accepted variables with a VIF < 10 (Montgomery et al., 2012).

To identify the main driving factors of the leaf microelement concentrations and BCFs, regression models using climate (aridity), human influence impact (HII) and soil variables (pH, EC, SOC and soil microelement concentrations) were generated based on Akaike Information Criterion (AIC), with leaf microelement concentrations and BCFs as response variables by the all-subset regression analysis. Best models were selected according to the lowest value of AIC, and the standardized coefficient of each factor was used to compare the relative importance of each in explaining the variations in the leaf microelement concentrations and BCFs. For both the leaf microelement concentrations and BCFs, aridity, HII, soil clay content, EC, SOC and soil pH were reserved (**Supplementary Tables 2, 3**; **Supplementary Figures 2, 3**). The analyses used the ‘leaps’ package in R (Lumley and Miller, 2020).

Structural equation models (SEMs) determined the relative importance of variables reserved by the all-subset regression for the leaf microelement concentrations and BCFs. The rationality of a causal model was examined, which was based on *priori* information on the relationships among the variables, the direct and indirect effects that one variable may have on another were partitioned, and the strengths of the multiple effects were estimated (Grace, 2006). Before modelling, the bivariate relationships between all variables were tested for linearity. The BCFs of Ni, Cu and Zn in leaves were affected curvilinearly by aridity, and these relationships were well described by a second-order polynomial (**Supplementary Figure 4**). To include polynomial relationships, the square of aridity was introduced into the model using a composite variable approach for the BCFs of leaf microelements. Then, *priori* models were established that were based on the known effects and relationships among leaf microelement concentrations and BCFs and were considered as factors (**Supplementary Figures 5, 6**). To test the *priori* model, the *χ* ^2^-test (the model has a good fit when 0 ≤ *χ* ^2^ ≤ 2 and 0.05 < *P* ≤ 1.00) and the root mean square error of approximation (RMSEA; the model has a good fit when 0 ≤ RMSEA ≤ 0.05 and 0.10 < *P* ≤ 1.00) were used. Since some variables were not distributed normally, the fit of the model was confirmed using the Bollen–Stine bootstrap test (the model has a good fit when 0.10 < bootstrap *P* ≤ 1.00) (Delgado-Baquerizo et al., 2013). The *priori* models provided a good fit to the data. To identify the dominant drivers for leaf microelement concentrations and BCFs, the direct and indirect effects of each variable affecting leaf microelement concentrations and BCFs and the total effect (summing the direct and indirect effects) for each variable were calculated. All SEM analyses used AMOS 21.0 (Amos Development Corporation, IBM SPSS, Chicago, IL, USA).

The functional threshold and indicator species of the leaf microelement concentrations and BCFs along environmental gradients were detected using threshold indicator taxa analysis (TITAN; Baker and King, 2010) with R package ‘TITAN2’. Indicator values (IndVal) of leaf microelement concentrations and BCF were first calculated for each plant species, which were obtained from the product of the relative concentrations and BCFs of microelements between groups and the frequency of occurrence of species within the group. The IndVal was used to identify the points of change in microelements and BCFs of species along the environmental gradient. The middle value of each two environmental gradients was then used as a candidate change point, and the gradients were iteratively divided into two groups. The environmental gradient corresponding to the maximum value of IndVal for each group on either side of the candidate change point was considered to be the threshold value for the species. The z-score was obtained by normalizing the IndVal score according to the mean and standard deviation, and, thus, the z-score distinguishes between negative (z-) and positive (z+) responses of each species to the environmental gradient (Baker and King, 2010). The cumulative values of z- and z+ scores for a species within a community assessed the negative and positive responses of the community to environmental gradients, respectively. The value of the environmental gradient corresponding to the maximum value is considered the threshold for the community (Baker and King, 2010). Species with less than three samples were not considered. Taxa leaf microelement concentrations and BCFs were log_10_(x+1)-transformed (Xiong et al., 2021).

## 3 Results

### 3.1 Leaf and soil microelement concentrations and leaf microelement BCFs

**Figure 2:**
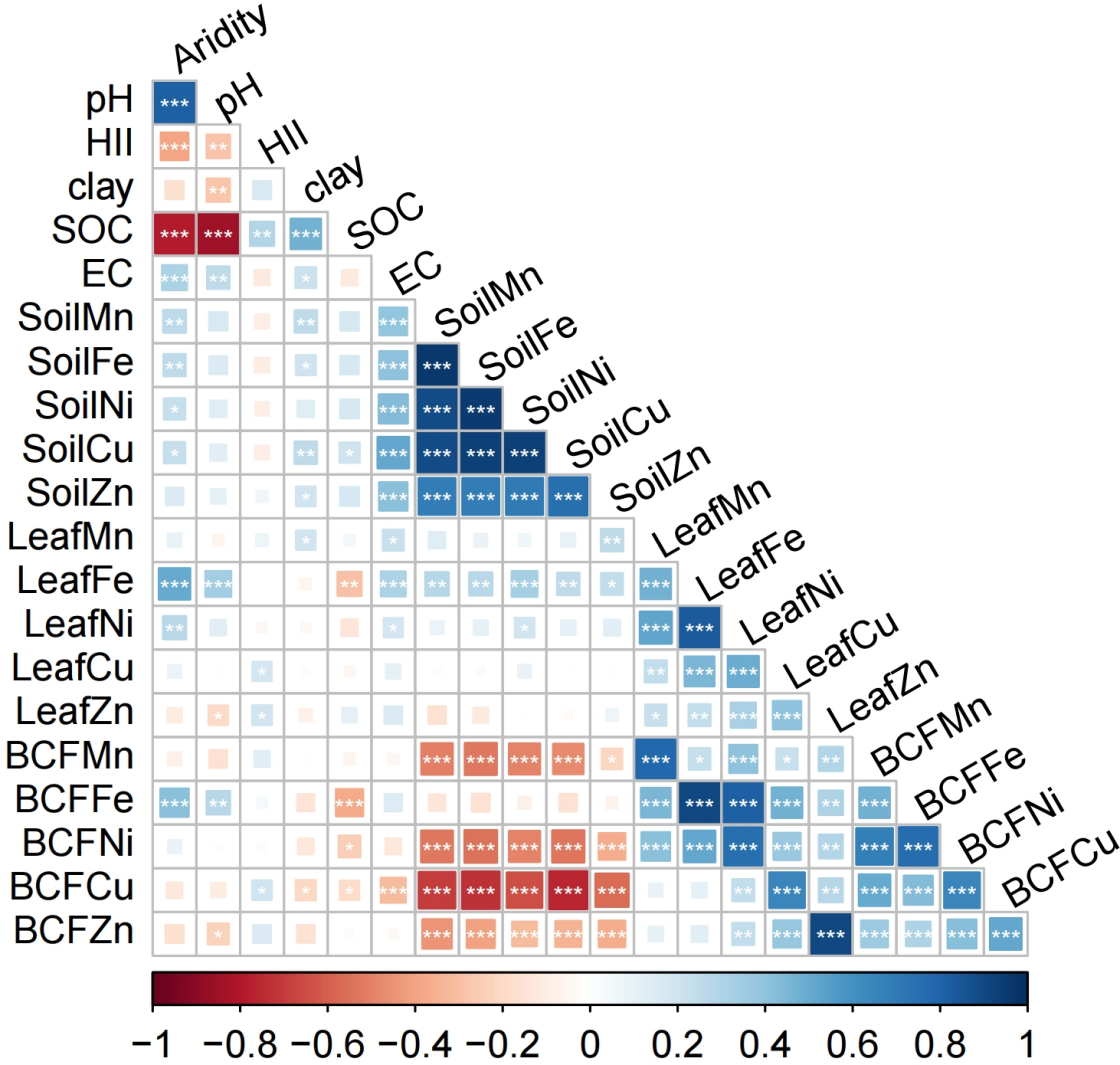
Pearson’s correlation matrix for aridity, HII, soil properties, leaf microelement concentrations and bioconcentration factors (BCFs). HII, human impact index; SOC, soil organic carbon; EC, soil electrical conductivity. Blue squares are positive correlations and red squares are negative correlations. *P< 0.05, **P< 0.01, ***P< 0.001.

Mean concentrations of the five microelements in leaves and soils varied greatly. Microelement concentrations ranged from 3.75E-5 mol/kg for Ni to 0.026 mol/kg for Fe in leaves, and from 2.99E-4 mol/kg for Cu to 0.51 mol/kg for Fe in soil. Leaf microelement BCFs ranged from 0.05 for Fe to 0.65 for Zn (**Table 1**; **Supplementary Figure 1**). There were significant positive relationships between soil microelement concentrations, as well as between leaf microelement concentrations and BCFs. Leaf microelement concentrations were correlated weakly with their respective soil microelement concentrations (**Figure 2**).

**Table 1:** Variations of leaf and soil total microelement concentrations and leaf microelement bioconcentration factors (BCFs).

	N	Mean	SE	Geo. mean	CV (%)	Minimum	Maximum
Leaf Mn (mol/kg)	111	2.13E-03	1.73E-04	1.66E-03	85.68	1.14E-04	0.011
Leaf Fe (mol/kg)	111	0.026	4.10E-03	0.013	166.3	1.14E-03	0.35
Leaf Ni (mol/kg)	111	3.75E-05	3.81E-06	2.74E-05	107.1	5.04E-06	2.67E-04
Leaf Cu (mol/kg)	111	1.26E-04	6.59E-06	1.11E-04	55	3.39E-05	3.84E-04
Leaf Zn (mol/kg)	111	4.85E-04	5.50E-05	3.18E-04	119.4	2.07E-06	4.20E-03
Soil Mn (mol/kg)	33	0.013	7.10E-04	0.011	32.26	1.25E-03	0.02
Soil Fe (mol/kg)	33	0.51	0.028	0.468	31.9	0.063	0.799
Soil Ni (mol/kg)	33	4.20E-04	3.13E-05	3.69E-04	42.88	4.54E-05	8.33E-04
Soil Cu (mol/kg)	33	2.99E-04	2.20E-05	2.64E-04	42.2	3.22E-05	5.63E-04
Soil Zn (mol/kg)	33	8.69E-04	5.97E-05	8.00E-04	39.45	2.65E-04	1.78E-03
BCF Mn	111	0.21	0.028	0.14	140.57	9.90E-03	2.55
BCF Fe	111	0.05	7.18E-03	0.028	148.72	2.62E-03	0.62
BCF Ni	111	0.11	0.011	0.074	105.02	9.90E-03	0.64
BCF Cu	111	0.61	0.075	0.42	130.8	0.077	4.83
BCF Zn	111	0.65	0.087	0.39	139.48	2.50E-03	5.69

Geo, Mean is the geometric mean; SE, standard error; CV, coefficient of variation.

### 3.2 Drivers of microelement concentrations and BCFs of leaves

Leaf microelement concentrations and BCFs of angiosperm species were combined to calculate the Blomberg’s *K*-value (**Supplementary Table 1**). The *K*-values were less than 1 and not significant, indicating that none of the traits revealed significant phylogenetic signals. Consequently, the influence of phylogeny was not considered in subsequent analyses on the microelement concentrations and BCFs.

**Figure 3:**
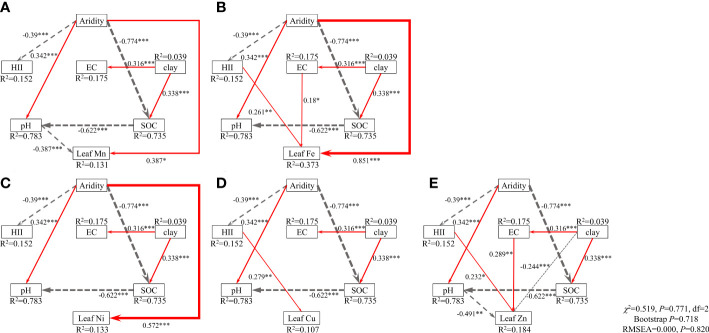
Effects of aridity, human impact index (HII), soil clay content, pH, electrical conductivity (EC) and organic carbon (SOC) on the concentrations of leaf Mn **(A)**, Fe **(B)**, Ni **(C)**, Cu **(D)** and Zn **(E)**. Numbers adjacent to arrows are standardized path coefficients (analogous to relative regression weights) and indicative of the effect size of the relationship. Continuous, red arrows indicate positive relationships, and dashed, grey arrows indicate negative relationships. The width of the arrows is proportional to the strength of path coefficients. R^2^ denotes the proportion of variance explained. Goodness-of-fit statistics for each model are shown in the lower right corner (df, degrees of freedom; RMSEA, root mean squared error of approximation), and all five of them are same. The priori model was refined by removing paths with non-significant relationships (see the priori model in **Supplementary Figure 5**). *P< 0.05, **P< 0.01, ***P< 0.001.

The SEM model explained 13.1%, 37.3%, 13.3%, 10.7% and 18.4% of the variance in the concentrations of Mn, Fe, Ni, Cu and Zn in leaves, respectively (**Figure 3**). Aridity affected the concentrations of leaf Mn, Fe and Ni positively (**Figures 3A–C**), while soil pH affected leaf Mn and Zn negatively (**Figures 3A, E**). Concentrations of leaf Fe, Cu and Zn were affected positively by HII (**Figures 3B, D, E**), and Fe and Zn concentrations were affected positively by EC (**Figures 3B, E**). The SEM explained 20.0%, 28.2%, 24.9%, 49.2% and 21.7% of the variance in the BCFs of Mn, Fe, Ni, Cu and Zn in leaves, respectively (**Supplementary Figure 7**). BCFs of Mn, Ni, Cu and Zn were affected negatively by soil pH and SOC (**Supplementary Figures 7A, C–E**), while the BCF of Fe was only affected negatively by soil pH (**Supplementary Figure 7B**). Aridity had negative effects on the BCFs of leaf Ni and Cu (**Supplementary Figures 7C, D**), but a positive effect on the BCF of leaf Fe (**Supplementary Figure 7B**).

**Figure 4:**
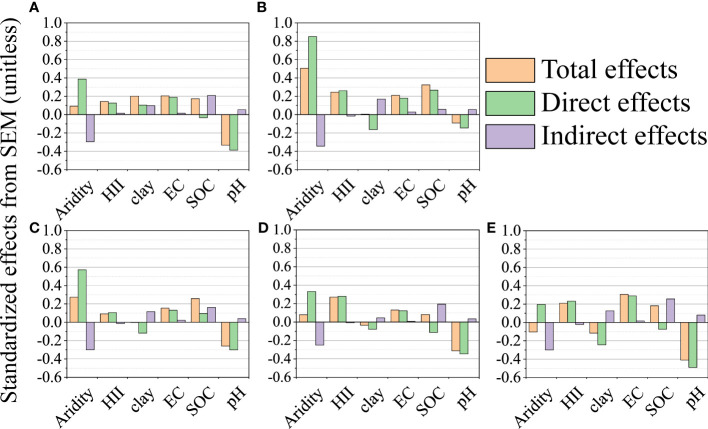
Total, direct and indirect effects based on the structural equation model (SEM) for the concentrations of leaf Mn **(A)**, Fe **(B)**, Ni **(C)**, Cu **(D)** and Zn **(E)**. Standardized total effects (direct plus indirect effects), and direct and indirect effects of aridity, human impact index (HII), soil clay content, pH, electrical conductivity (EC) and organic carbon (SOC) on the concentrations of leaf Mn, Fe, Ni, Cu and Zn. The yellow column is the total effect, the green column is the direct effect, and the purple column is the indirect effect.

The direct and indirect drivers of the microelement concentrations and BCFs in leaves were identified by SEM (**Figure 4**; **Supplementary Figure 8**). Aridity had the greatest direct positive effect and soil pH had the greatest direct negative effect on leaf microelement concentrations. SOC and EC were strong drivers of leaf microelement concentrations (**Figure 4**). Aridity had a direct, slight, negative effect on BCF of leaf Mn, a strong, positive effect on the BCF of leaf Fe, and an indirect, negative effect on BCFs of leaf Ni, Cu and Zn. Soil pH and SOC had strong, direct, negative effects on BCFs of leaf Mn, Fe, Ni, Cu and Zn (**Supplementary Figure 8**). HII and clay content had minor effects on both leaf microelement concentrations and BCFs.

### 3.3 Threshold levels and indicators along environmental gradients

**Figure 5:**
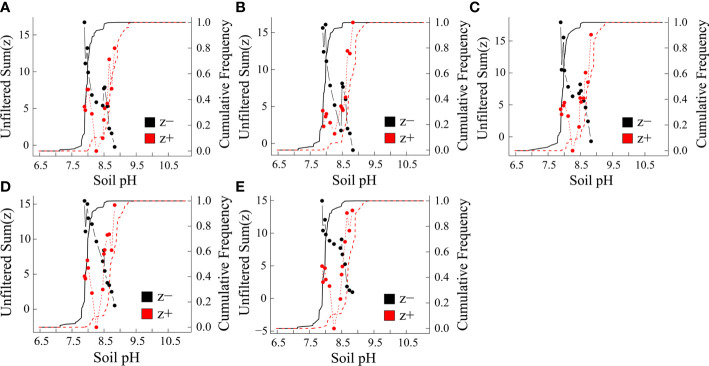
Leaf microelement concentrations and bioconcentration factors (BCFs) in response to soil pH. **(A)** Mn **(B)** Fe **(C)** Ni **(D)** Cu and **(E)** Zn. Black and red symbols present accumulative negative (sum z-) and positive (sum z+) responses, respectively. Vertical dashed lines indicate pH gradient thresholds corresponding to maximal sum (z).

Environmental thresholds of microelement concentrations and BCFs were determined in leaves by cumulating z− and z+ change points. Aridity, soil pH and SOC had the strongest impacts on leaf microelement concentrations and BCFs, and, therefore, the thresholds and indicator species along aridity, soil pH and SOC gradients were assessed further. Based on TITAN, both leaf microelement concentrations and BCFs did not differ significantly along the SOC gradient, so they are not presented. There was an obvious peak for the leaf microelement concentrations and BCFs along the aridity gradient for both sum (z-) and sum (z+) at 0.89 (**Supplementary Figure 9**), and along the soil pH gradient at 7.9 and 8.9 (**Figure 5**).

**Figure 6:**
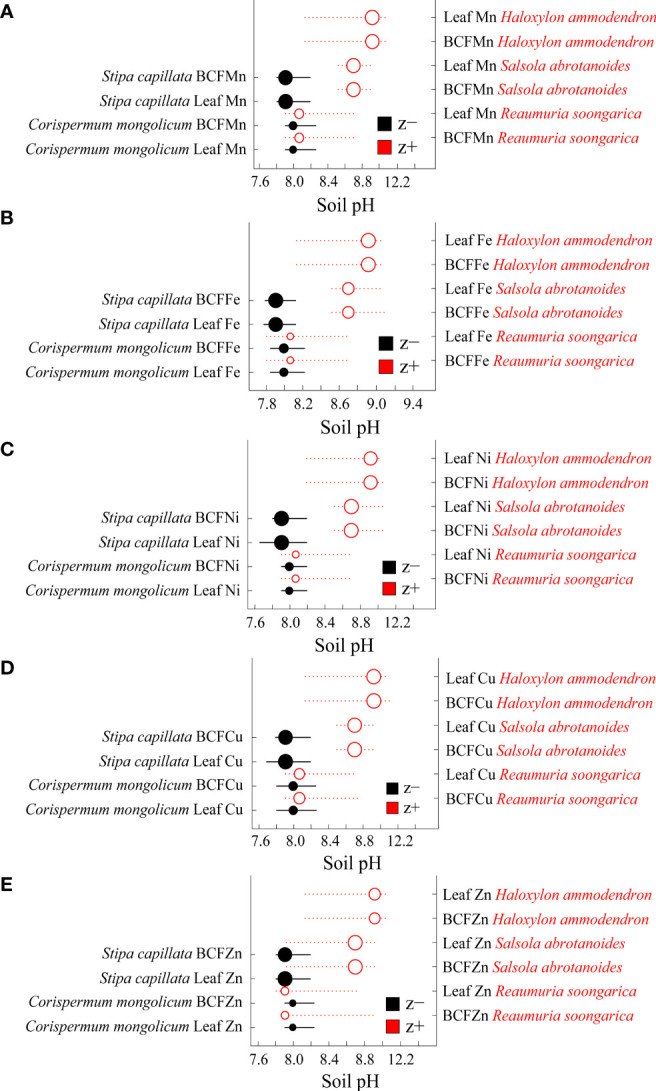
Threshold Indicator Taxa Analysis (TITAN) of leaf microelement concentrations and bioconcentration factors (BCFs) in response to soil pH. **(A)** Mn and the BCF of Mn, **(B)** Fe and the BCF of Fe, **(C)** Ni and the BCF of Ni, **(D)** Cu and the BCF of Cu and **(E)** Zn and the BCF of Zn. Black and red symbols represent accumulative negative (sum z-) and positive (sum z+) responses, respectively. Symbol size is in proportion to z scores (magnitude of response). Horizontal lines represent the 95% bootstrap confidence intervals. Woody and herbaceous species are shown in red and in black fonts, respectively.

For the aridity gradient, z− of leaf microelement concentrations and BCFs were the woody species *Artemisia halodendron* and the herbaceous species *Stipa capillata* and *Corispermum mongolicum*, and the z+ for the woody species *Reaumuria soongorica* (**Supplementary Figure 10**). For soil pH, all z− were herbaceous species (*Stipa capillata* and *Corispermum mongolicum*), and all z+ were woody species (*Haloxylon ammodendron*, *Salsola abrotanoides* and *Reaumuria soongorica*) (**Figure 6**).

## 4 Discussion

### 4.1 Leaf and soil microelement concentrations and BCFs in leaves

In the present study, the concentrations of leaf and soil Mn, Fe, Ni, Cu and Zn and the leaf BCFs of these elements were determined in the drylands of northern China. Leaf microelement concentrations were within the normal range of mature leaf microelements [CNEMC (China National Environmental Monitoring Centre), 1990], which supported our first hypothesis. The concentration of leaf Mn was similar to leaf Fe concentration, but was slightly greater than concentrations in leaves in Chinese terrestrial plants (Han et al., 2011), while Mn, Ni, Cu and Zn concentrations in leaves were similar to leaf Fe concentration, but were slightly greater than concentrations in leaves in forests of eastern China (Zhao et al., 2016). Leaf Mn, Fe, Cu and Zn concentrations were similar to those reported in the dominant herbaceous plant species in the Alxa Desert (He et al., 2016).

The soil total microelement concentrations in the present study were generally below the national Chinese levels [CNEMC (China National Environmental Monitoring Centre), 1990]. Concentrations of Mn, Ni, Cu and Zn were on average lesser and of leaf Fe concentration was greater than concentrations of these elements in six land use types in southeast China (Ren et al., 2019). In addition, soil Fe, Cu and Zn concentrations were similar to the soil Mn concentration, but was lesser than concentrations in the Taklamakan Desert (Zhang et al., 2022), while soil Mn and Zn concentrations were greater and of soil Fe and Zn concentrations were similar to soil concentrations in the Alxa Desert (He et al., 2016).

Significant correlations emerged among soil total microelement concentrations, probably because of the strong sorption capacity of microelement oxides to other microelements. For example, microelements in soil can be adsorbed and co-precipitated with Mn/Fe oxides, which leads to increased correlations among soil microelement concentrations (Suda and Makino, 2016). There were also significant positive relationships among the leaf microelement concentrations and BCFs. This occurrence might be due, at least in part, to the positive synergistic interactions among leaf microelements (Kabata-Pendias, 2011). The weak relationships between leaf and soil microelement concentrations may be the result of the dilution effect of plants in water-limited ecosystems. Increasing aridity may result in a greater element uptake by the plant than needed, making the plant less dependent on soil elements (Luo et al., 2016).

Desert plants rely on internal nutrient cycling and require mineral elements within specific ranges for optimal growth and functions (Sardans et al., 2015; Huang et al., 2018). These plants possess a certain degree of stoichiometric flexibility to respond to the stress of deficient water and soil nutrients in drylands (He et al., 2016). The homeostasis strategy of plants to maintain normal nutrient ranges enables their growth even under nutrient stress (Sterner and Elser, 2002).

### 4.2 Factors influencing leaf concentrations and bioconcentration factors of microelements in drylands

The all-subset regression analysis and structural equation modeling indicated that leaf microelement concentrations and BCFs were regulated by aridity, HII, and soil physical properties, which partially supported our second hypothesis. Among these variables, aridity, soil pH and SOC were the strongest drivers of both leaf microelement concentrations and BCFs. The large direct positive effect of aridity on leaf Mn, Fe, Ni, Cu and Zn concentrations could be attributed to the low concentration of soil microelements compared with the background value. Due to concentration effects and regulation of plant growth equilibrium, nutrient concentrations do not decrease when plants grow slower under drought, even with reduced nutrient absorption (Sardans and Peñuelas, 2004). Furthermore, interactions between shrubs and herbs may result in different responses to drought. In more xeric sites, the deep roots of shrubs could mitigate drought stress through hydraulic and nutrient lift effects, and, thus, increase nutrient availability in the topsoil for neighboring herbs. This promotes a greater uptake of soil nutrients by herbaceous plants, resulting in increased nutrient concentrations (Wang et al., 2019).

The current study provided empirical evidence that aridity reduces BCFs of leaf Mn, Ni, Cu and Zn, but increases the BCF of Fe. How can these patterns be explained? Drought can reduce element uptake of plants by reducing mineralization or decreasing the diffusion and flow rates of soil elements (He and Dijkstra, 2014). However, with aridity-induced water deficit and high soil temperature, ferrihydrite (Fe (OH)_2_) and goethite (α-FeOOH) are converted to haematite (α-Fe_2_O_3_), that is, a form of Fe that is more recalcitrant and less mobile in soils (Moreno-Jiménez et al., 2019), resulting in reduced availability of soil Fe. With a deficiency in soil Fe, dicotyledonous and non-gramineous plants and gramineous plants use two different strategies to chelate Fe ions from the soil. Dicotyledonous and non-gramineous plants obtain Fe ions by acid-solubilizing and reducing Fe^3+^ from the rhizosphere and then absorbing Fe^2+^ into the cells through a high-affinity Fe transport system (strategy I). In contrast, gramineous plants obtain Fe ions by absorbing Fe^3+^ from soil on the basis of chelation (strategy II) (Marschner, 2012).

Among the soil properties that affect the uptake of plant elements, soil pH plays the most important role in determining element speciation, metal surface solubility and migration, and, ultimately, metallic element bioavailability (Zeng et al., 2011; Fijałkowski et al., 2012). The pH mediates acid-base equilibrium directly, and affects the complexation of metal cations and ion exchange in soil (Janoš et al., 2010). Microelements are generally easiest available to plants in a pH range of 5 to 7, and less so outside this optimal range (McCauley et al., 2009). The pH in the present study was greater than 7 in most areas. A high soil pH causes metals to precipitate as hydroxides and combine preferentially with ionized functional groups of solid soil components (Sposito, 2008; Hamid et al., 2020). A decrease in metal solubility reduces the content of available metallic elements in the soil, restricts plant absorption of elements (Sacristán et al., 2015), and, thereby, reduces the leaf microelement concentrations and BCFs.

SOC also affected soil metal availability and mobility, as organic matter: (1) provides organic chemicals to soil solutions, which are used as chelates to increase the utilization of metals by plants; and (2) retains more metals by adsorption or forming stable complexes with humus, resulting in an increase in total metal content and decrease in metal availability in soil (Zeng et al., 2011; Niesiobedzka, 2012). Humus can fix metals on solid materials in an arid soil environment (Chen et al., 2016), which could partially explain the negative correlation of leaf BCFs and SOC in the current study. In addition, organic matter has high selectivity for cations of microelements (Kwiatkowska-Malina, 2018), and most of the transition cations (Pb, Cu, Cr, Ni, Co, Zn, Al, Fe and Mn) are adsorbed to a greater extent than alkali metal cations (Violante et al., 2010). As a result, organic matter retains more microelements and limits the absorption of microelements by plants (BCFs).

Interestingly, the effect of EC (an approximate representation of soil salinity) was stronger on leaf microelement concentrations than on the BCFs. A possible mechanism for the stronger direct and positive effects of EC on leaf microelement concentrations is that salinity causes an increase in reactive oxygen species (ROS), with deleterious effects on cell metabolism. However, ROS is usually detoxified by superoxide dismutases (SODs) containing Mn, Fe, Cu or Zn metals for survival in saline soils (Slater et al., 2008; Patel et al., 2009). Additionally, the relatively weak direct and positive effects of anthropogenic activities on leaf microelement concentrations indicate that these activities increase the metallic elements released into soil, water and atmosphere and, thus, enhance leaves absorbing and accumulating microelements (Kabata-Pendias, 2011). The small effect of clay content on both the leaf microelement concentrations and BCFs may be due to the coarse texture and poor development of dryland soils (Dregne, 1976), which minimize soil weathering and the formation of active sites on minerals, thereby, reducing their influence on metal availability (Moreno-Jiménez et al., 2019).

### 4.3 Thresholds and indicators of leaf microelement concentrations and BCFs with aridity and soil pH gradients

Soil pH and SOC (as also reported by Hu et al., 2020) and aridity were key environmental variables in the absorption and accumulation of plant elements in the present study. We then determined ecological thresholds and indicator species for the leaf microelement concentrations and BCFs along gradients of these variables. In support of our third hypothesis, there were abrupt shifts of leaf microelement concentrations and BCFs along the aridity and soil pH gradients, with a key tipping point for aridity at 0.89, and key tipping points for soil pH at 7.9 and 8.9. However, there was no obvious tipping point along the SOC gradient. Supporting our fourth hypothesis, indicator species for the soil pH gradient were clearly separated between herbaceous and woody species: leaf microelement concentrations and BCFs of woody species (*Haloxylon ammodendron*, *Salsola abrotanoides* and *Reaumuria soongorica*) and herbaceous species (*Stipa capillata* and *Corispermum mongolicum*) were positive and negative indicators, respectively. Along the aridity gradient, *Reaumuria soongorica* was a positive indicator, while *Artemisia halodendron*, *Stipa capillata* and *Corispermum mongolicum* were negative indicators.

Soil pH, aridity and EC were correlated positively, which indicates that areas with high aridity also had high soil alkalinity and salinity. To cope with drought, desert woody plants tend to allocate more photosynthate to roots than shoots. This results in higher root: shoot ratios and enables the plant to absorb more water and minerals (Chen et al., 2019). Moreover, desert plant species have other distinct adaptive strategies for survival (Akram et al., 2020; Yao et al., 2021a). For example, *Haloxylon ammodendron* developed water storage tissue in assimilating branches to increase water retention and water absorption capabilities and, thus, to better tolerate drought (Gong et al., 2019). Crystal cells in water storage tissue not only alter the osmotic pressure of the cells, but also accumulate excess salt to avoid salt toxicity (Grigore and Toma, 2021). *Reaumuria soongorica* possesses salt glands on the leaf surface, which secrete excess ions, regulate ion balance and maintain osmotic pressure stable to minimize drought and salt-alkaline stress (He et al., 2019). Consequently, shrub species can serve as positive indicators of aridity and soil pH.

The herbaceous plant species *Stipa capillata* is widespread in Eurasia and is distributed in montane steppe where only it can be dominant (Li et al., 2020). *Corispermum mongolicum* is a typical sandy species distributed widely in deserts, and acts mainly as a pioneering species in sandy arid environments (Wang et al., 2009). Herbaceous plant species are sensitive to soil biota (Kulmatiski et al., 2008), and, in comparison to woody plant species, tend to grow faster but have higher nutrient requirements (Poorter, 1989; Gong et al., 2020). These species act primarily as positive indicators for soil metallic elements and nutrients, and as negative indicator for aridity and soil pH (Xiong et al., 2021). In addition, some herbaceous plant species have inherent characteristics that cause them to exhibit a weaker ability to resist stress than woody plant species. For example, *Corispermum mongolicum* acts as a mesophyte, and has a thin leaf cuticle and poorly developed xeromorphic structures, which results in a high transpiration rate, a low water holding capacity (Wang et al., 2004), and poor resistance to water deficits.

## 5 Conclusions

Our results demonstrated that leaf microelement concentrations can be maintained within the normal ranges to satisfy growth requirements, regardless of their concentrations in soil (homeostasis), which supports our first hypothesis. Supporting our second hypothesis, leaf microelement concentrations and BCFs were regulated by aridity, HII and soil properties, where aridity, soil pH and SOC were the main drivers of both the microelement concentrations and BCFs of leaves, while EC was also important for leaf micronutrient concentrations. In support of our third hypothesis, there were shifts in leaf microelement concentrations and BCFs at an aridity of 0.89 and soil pHs of 7.9 and 8.9. These results suggest that leaf microelement concentration and BCF thresholds, combined with local aridity and soil pH, can be used as predictors of plant responses to future climate change. Finally, as hypothesized, woody species, with a strong tolerance of arid and alkaline environments, were positive indicators of leaf microelement concentrations and BCFs, while herbaceous species, with a lesser tolerance of arid and alkaline environments, were mainly negative indicators along aridity and soil pH gradients. The identification of indicator species for leaf microelement concentrations and BCFs along environmental gradients can be beneficial in understanding species-specific biogeochemical processes and adaptation strategies of dryland plants.

## Data availability statement

The original contributions presented in the study are included in the article/**Supplementary Material**. Further inquiries can be directed to the corresponding authors.

## Author contributions

JR, JX and JD designed this study. Field observational data were obtained by all authors. All authors carried out the laboratory measurements and data analyses. YZ, JR, JX and JD wrote the first draft of the manuscript and AD edited the final version. All authors contributed critically to the drafts and gave final approval for publication. All authors contributed to the article and approved the submitted version.
