# Supplementary material for: Concentrations and bioconcentration factors of leaf microelements in response to environmental gradients in drylands of China
Source: Front Plant Sci. 2023 Mar 2;14:1143442. doi: 10.3389/fpls.2023.1143442 (PMC10019776; doi:10.3389/fpls.2023.1143442)
Supplement: Supplementary file 1 [file DataSheet_1.docx]

**Supplementary Table 1 Phylogenetic signals of different traits with microelement concentrations and bioconcentration factors (BCFs) of leaf in dominant plants**

|  | **Phylogenetic signal statistics** | |
| --- | --- | --- |
| **Trait** | ***K*-value** | ***P*-value** |
| **Leaf Mn** | 0.094 | 0.528 |
| **Leaf Fe** | 0.134 | 0.162 |
| **Leaf Ni** | 0.112 | 0.370 |
| **Leaf Cu** | 0.052 | 0.863 |
| **Leaf Zn** | 0.102 | 0.502 |
| **BCF of leaf Mn** | 0.117 | 0.300 |
| **BCF of leaf Fe** | 0.109 | 0.331 |
| **BCF of leaf Ni** | 0.100 | 0.416 |
| **BCF of leaf Cu** | 0.102 | 0.413 |
| **BCF of leaf Zn** | 0.101 | 0.503 |

**Supplementary Table 2** **Summary of all-subset regression models with the leaf microelement concentrations as the dependent variables, and aridity, human impact index (HII), soil clay content, pH, electrical conductivity (EC), organic carbon (SOC) and soil microelement concentrations as the independent variables.**

| **Formula** | **AIC** | ***r^2^*** | ***P*** |
| --- | --- | --- | --- |
| **Leaf Mn=-2.58+0.68Aridity+0.009HII-0.13pH+0.09EC** | 49.19 | 0.087 | <0.01 |
| **Leaf Fe=-3.86+2.10Aridity+0.02HII-0.01clay+0.11EC+0.31SOC** | 118.23 | 0.34 | <0.001 |
| **Leaf Ni=-4.5+0.83Aridity-0.10pH+0.05EC** | 58.68 | 0.09 | <0.01 |
| **Leaf Cu=-4.02+0.44Aridity+0.01HII-0.05pH** | -28.31 | 0.06 | <0.05 |
| **Leaf Zn=-2.90+0.02HII-0.01clay-0.12pH+0.17EC** | 111.66 | 0.13 | <0.001 |

**Supplementary Table 3** **Summary of all-subset regression models with the microelement bioconcentration factors (BCFs) of leaf as the dependent variables, and aridity, human impact index (HII), soil clay content, pH, electrical conductivity (EC) and organic carbon (SOC) as the independent variables.**

| **Formula** | **AIC** | ***r^2^*** | ***P*** |
| --- | --- | --- | --- |
| **BCF Mn=1.23+0.01clay-0.30pH-0.53SOC** | 70.65 | 0.16 | <0.001 |
| **BCF Fe=-1.45+1.45Aridity+0.02HII-0.22pH-0.32SOC** | 126.07 | 0.24 | <0.001 |
| **BCF Ni=0.72-0.26pH-0.53SOC** | 74.7 | 0.17 | <0.001 |
| **BCF Cu=1.58-0.42Aridity+0.01HII-0.23pH-0.07EC-0.60SOC** | 22.92 | 0.36 | <0.001 |
| **BCF Zn=1.90+0.01HII-0.33pH-0.50SOC** | 123.51 | 0.15 | <0.001 |


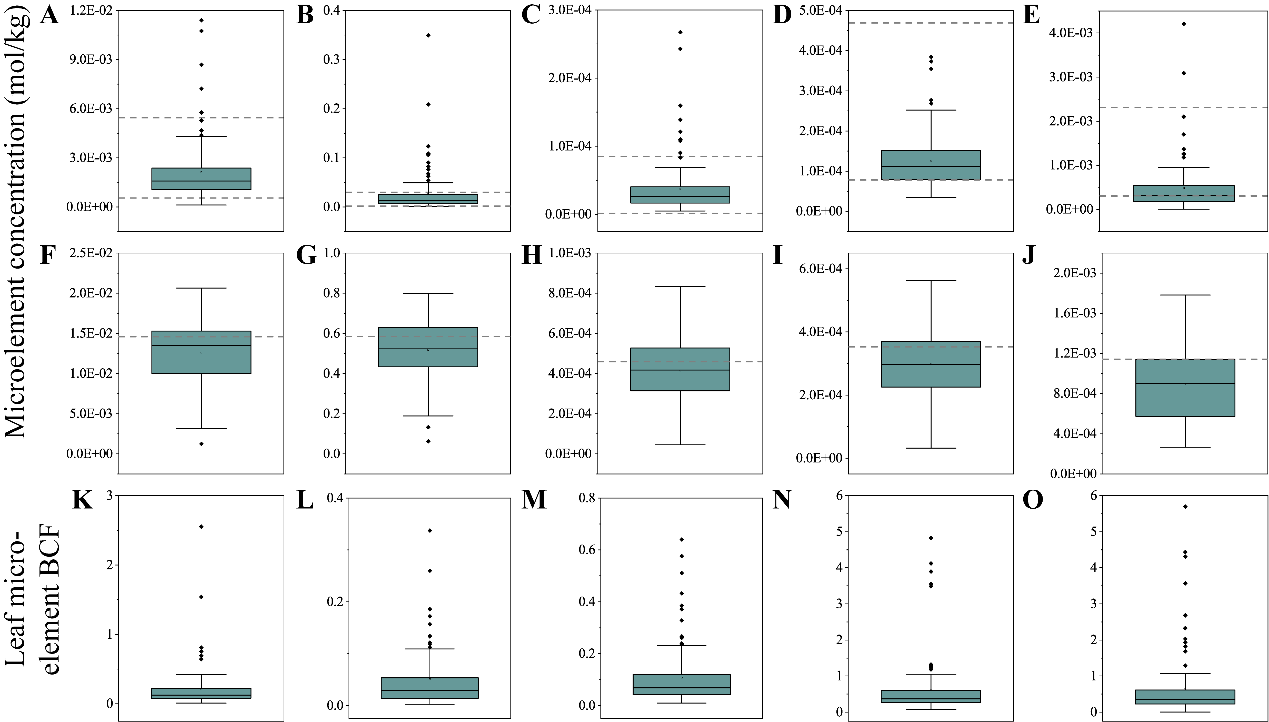


**Supplementary Figure 1 Box plots of microelement (Mn, Fe, Ni, Cu and Zn) concentrations of leaves (A - E) and soils (F - J) and leaf microelement BCFs (K - O).** Dashed lines indicate the normal range of microelement concentrations in mature leaves (Kabata-Pendias, 2011) and the national average level in soils (CNEMC 1990).

**References**

CNEMC (China National Environmental Monitoring Centre). 1990. *Background Values of Soil Elements in China*, Beijing: Environmental Science Press.

Kabata-Pendias, A. (2011). *Trace elements in soils and plants, Fourth Edition*. Boca Raton: CRC press. doi: 10.1201/b10158


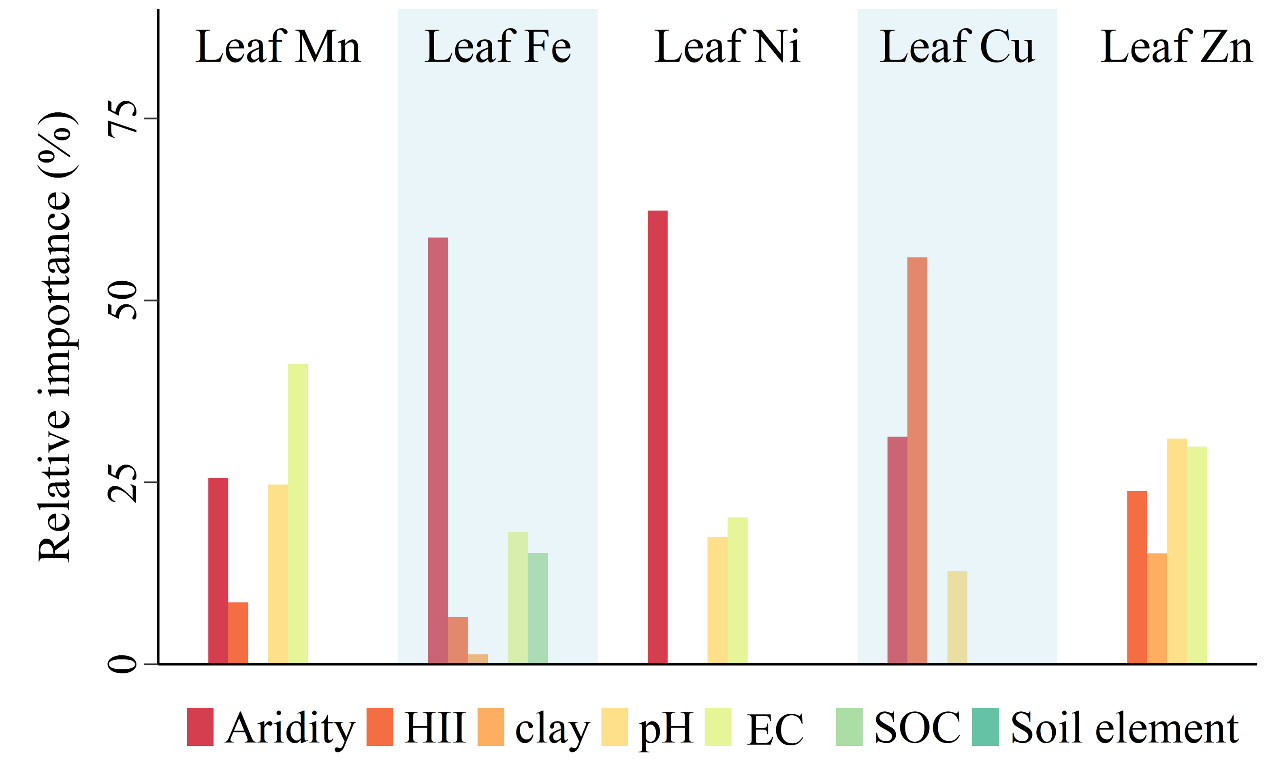


**Supplementary Figure 2 The relative importance of aridity, human impact index (HII), soil clay content, pH, electrical conductivity (EC), organic carbon (SOC) and concentrations of soil microelements (Mn, Fe, Ni, Cu and Zn) on the leaf microelement concentrations from the all-subset regression models.** Relative importance is the contribution of each predictor to the R^2^.


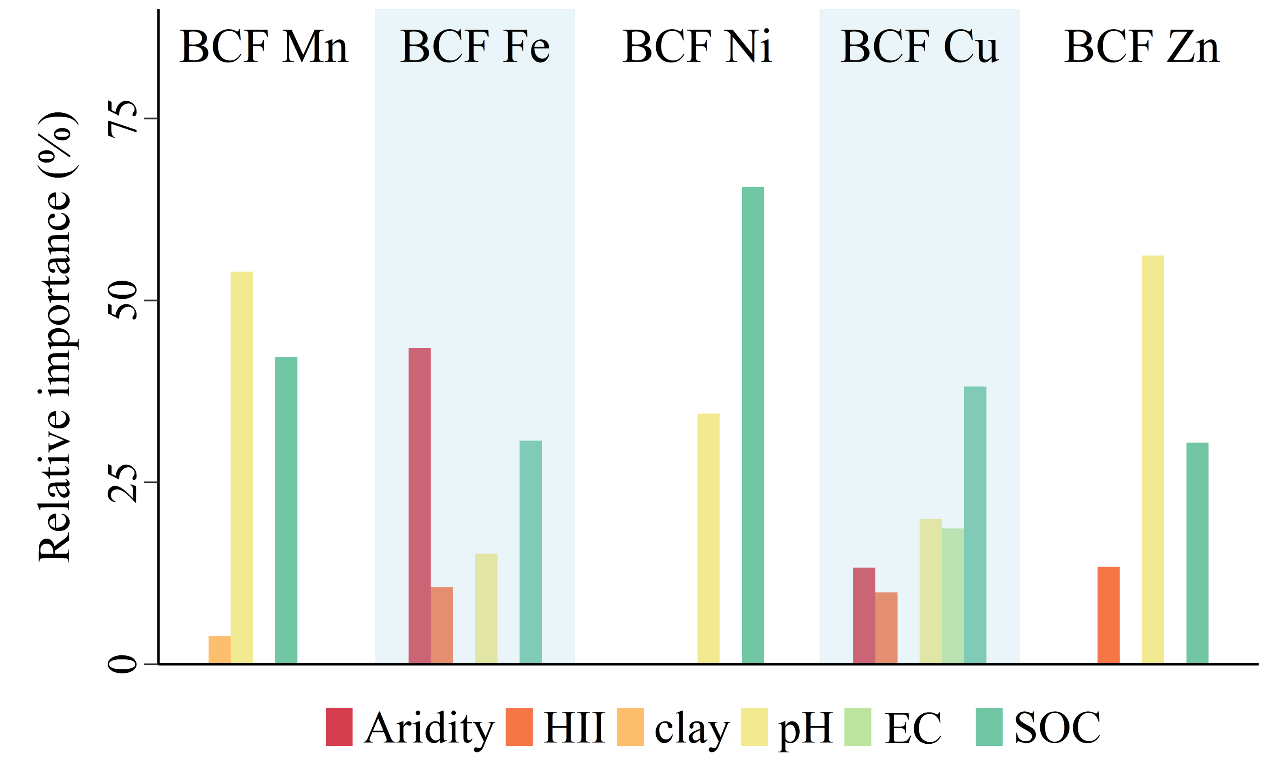


**Supplementary Figure 3 The relative importance of aridity, human impact index (HII), soil clay content, pH, electrical conductivity (EC) and organic carbon (SOC) for the plant microelement bioconcentration factors (BCFs) of leaf from the all-subset regression models.** Relative importance is the contribution of each predictor to the R^2^.


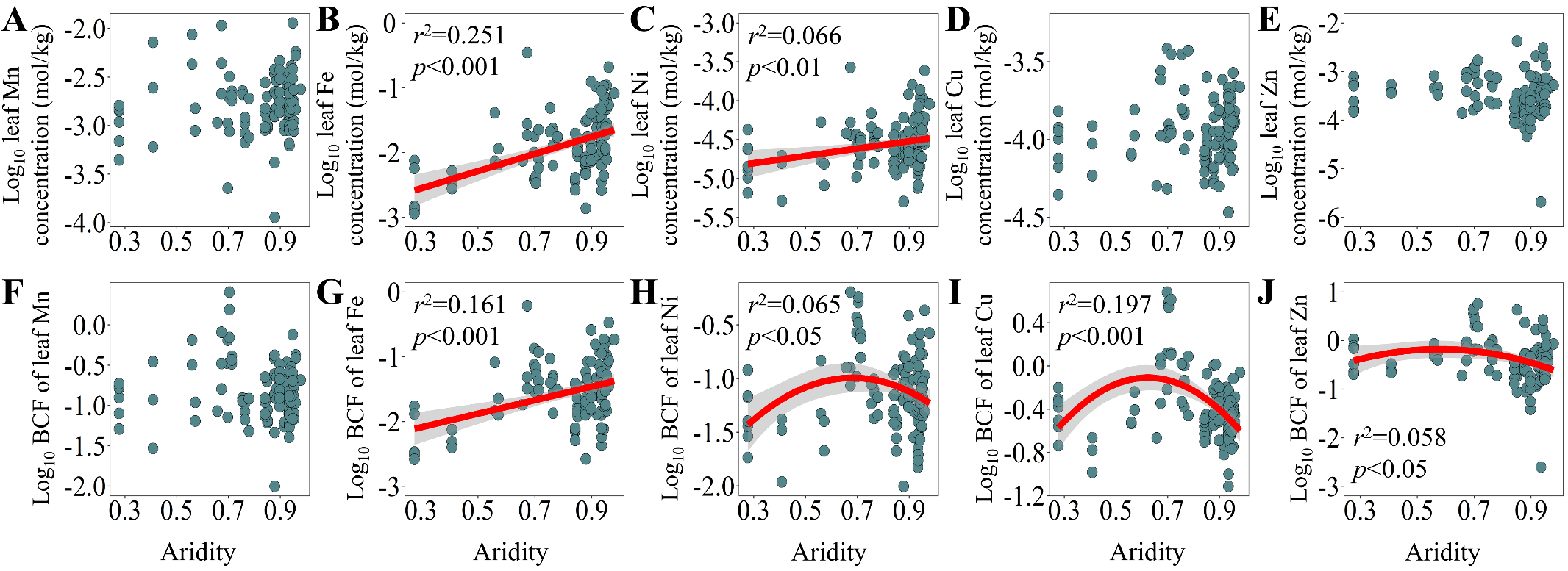


**Supplementary Figure 4 Trends in leaf microelement concentrations and bioconcentration factors (BCFs) along an aridity (1-AI) gradient.** (A) – (E) describe the microelement (Mn, Fe, Ni, Cu and Zn) concentrations in leaves. (F) – (J) describe the BCFs of leaf microelements. The regression line is presented only when significant (*P* < 0.05).


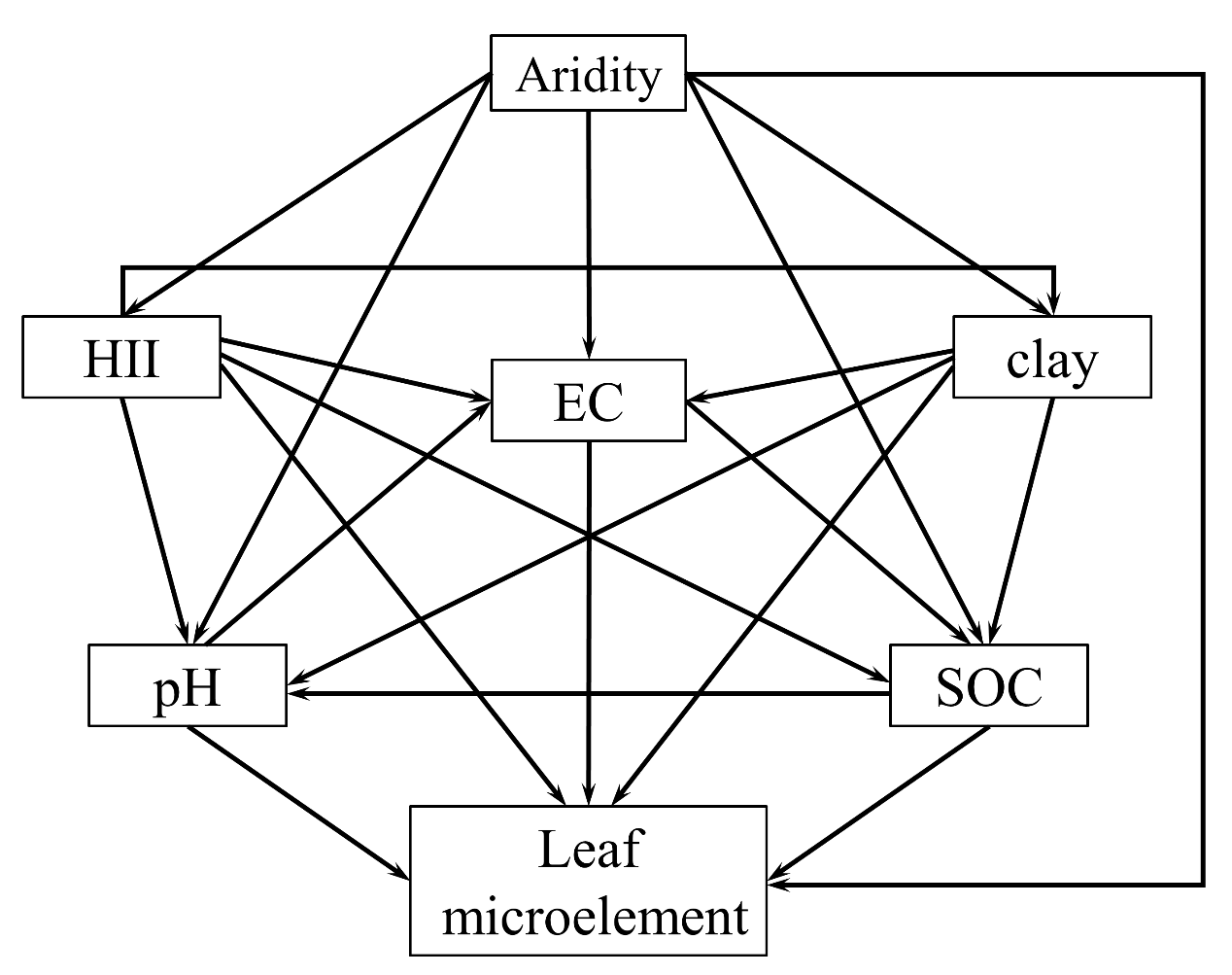


**Supplementary Figure 5 The *priori* structural equation model (SEM) for the concentrations of leaf microelements (Mn, Fe, Ni, Cu and Zn).** Aridity, human impact index (HII), soil clay content, pH, electrical conductivity (EC) and organic carbon (SOC) were included as predictors. Differences between the *priori* and final model structures were due to the removal of non-significant paths.


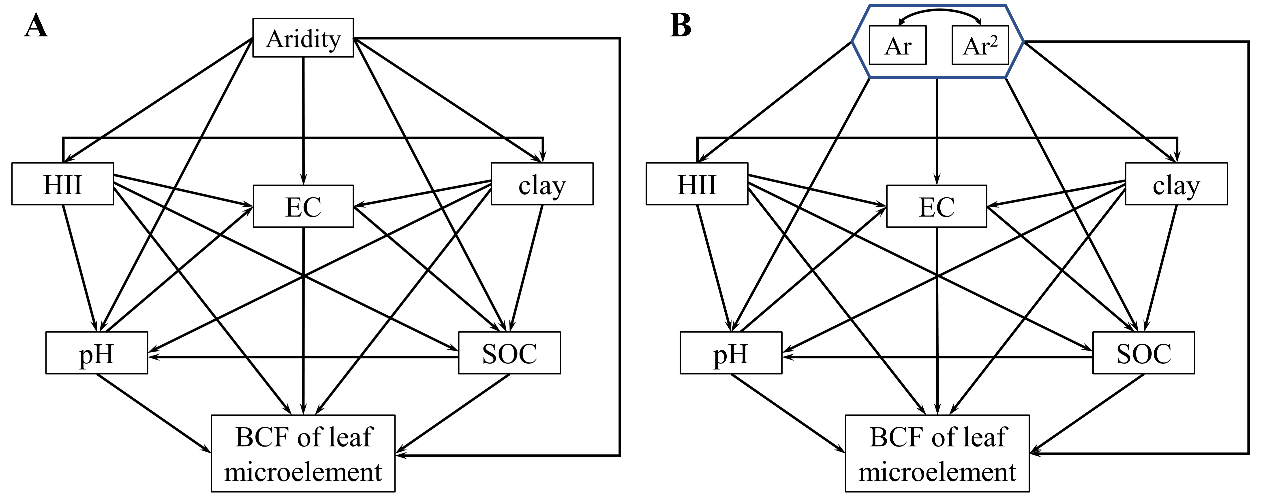


**Supplementary Figure 6 The *priori* structural equation models (SEMs) for the plant bioconcentration factors (BCFs) of leaf microelements (Mn, Fe, Ni, Cu and Zn).** (A) was the BCFs of Mn and Fe, and (B) was the BCFs of Ni, Cu and Zn. Aridity (composite variable formed from Ar and Ar^2^), human impact index (HII), soil clay content, pH, electrical conductivity (EC) and organic carbon (SOC) were included as predictors. Differences between the *priori* and final model structures were due to the removal of non-significant paths.


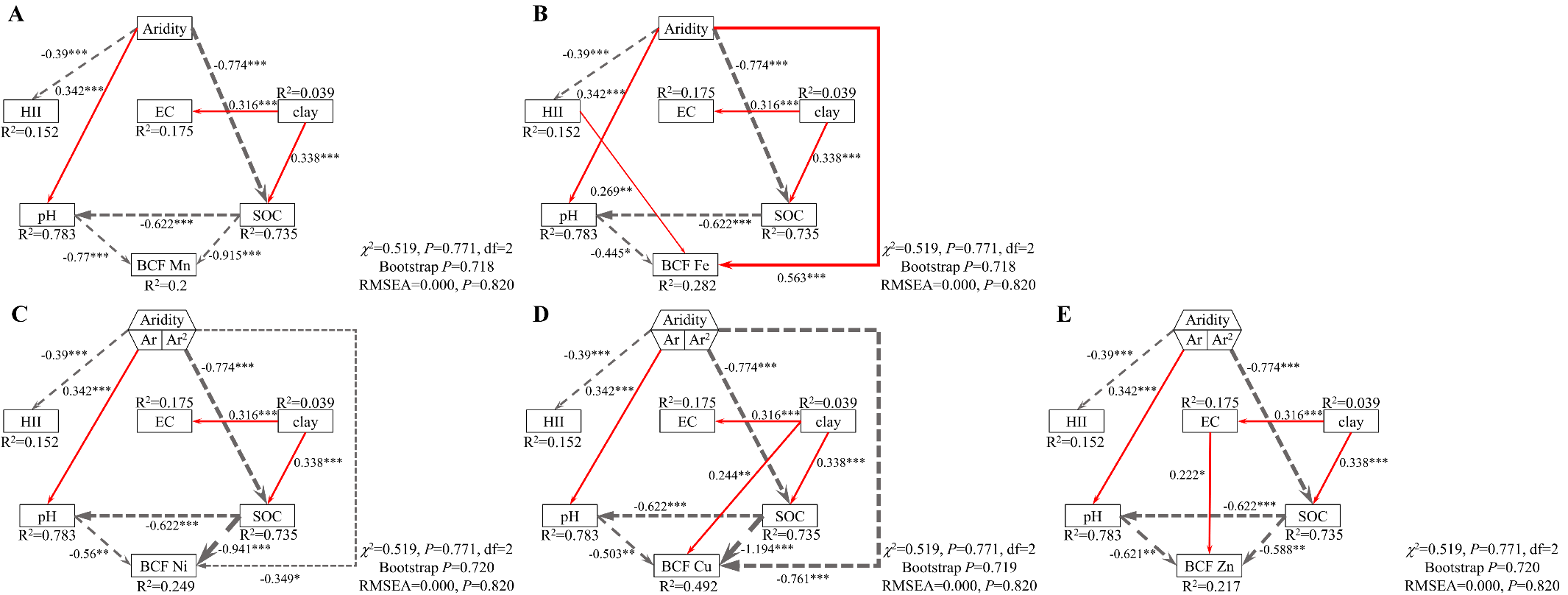


**Supplementary Figure 7** **Effects of aridity, human impact index (HII), soil clay content, pH, electrical conductivity (EC) and organic carbon (SOC) on the bioconcentration factors (BCFs) of leaf Mn (A), Fe (B), Ni (C), Cu (D) and Zn (E).** Numbers adjacent to arrows are standardized path coefficients (analogous to relative regression weights) and indicative of the effect size of the relationship. Continuous, red arrows indicate positive relationships, and dashed, grey arrows indicate negative relationships. The width of the arrows is proportional to the strength of path coefficients. R^2^ denotes the proportion of variance explained. Goodness-of-fit statistics for each model are shown in the lower right corner (df, degrees of freedom; RMSEA, root mean squared error of approximation). The *priori* model was refined by removing paths with non-significant relationships (see the *priori* model in Supplementary Figure 6). **P* < 0.05, ***P* < 0.01, ****P* < 0.001.


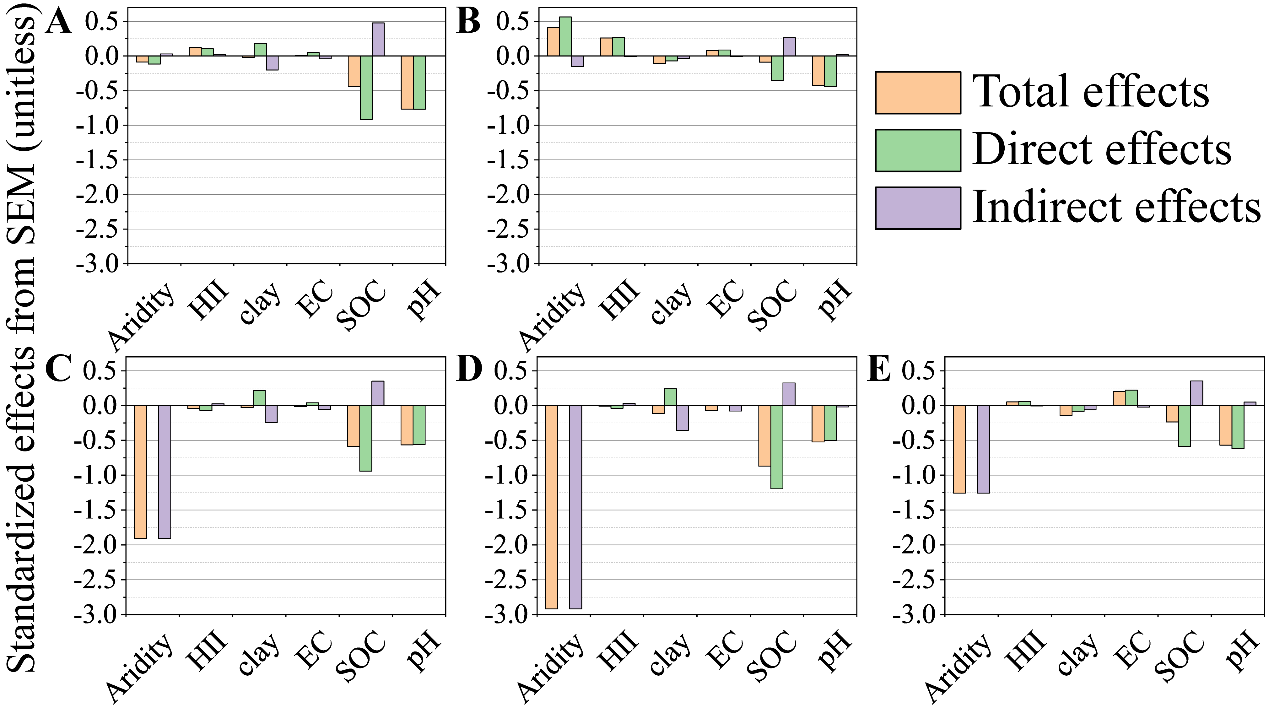


**Supplementary Figure 8 Total, direct and indirect effects based on the structural equation model (SEM) for the leaf microelement bioconcentration factors (BCFs).** Standardized total effects (direct plus indirect effects), and direct and indirect effects of aridity, human impact index (HII), soil clay content, pH, electrical conductivity (EC) and organic carbon (SOC) on the BCFs of leaf Mn (A), Fe (B), Ni (C), Cu (D) and Zn (E). The yellow column is the total effect, the green column is the direct effect, and the purple column is the indirect effect.

**
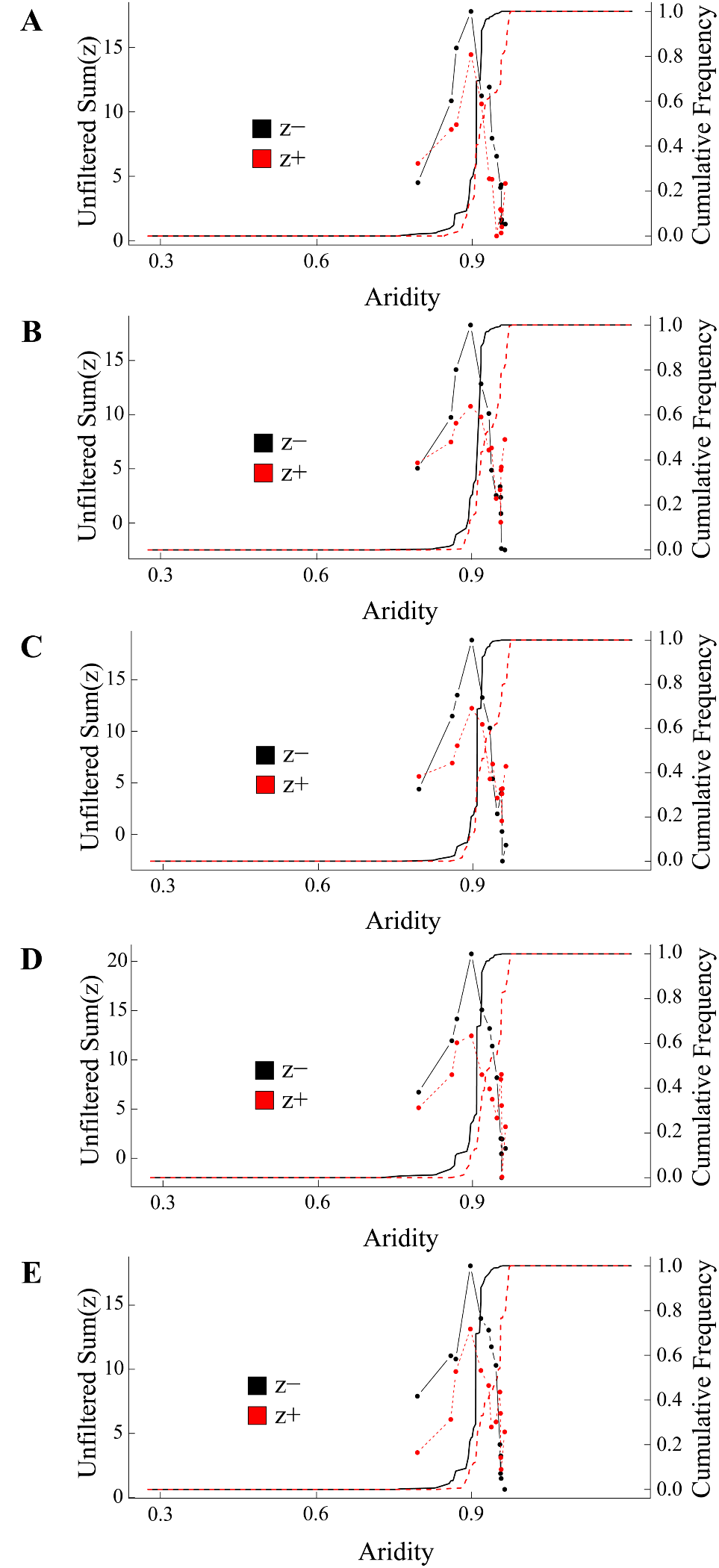
**

**Supplementary Figure 9 Leaf microelement concentrations and bioconcentration factors (BCFs) of leaf microelements in response to aridity.** (A) Mn, (B) Fe, (C) Ni, (D) Cu and (E) Zn. Black and red symbols denote accumulative negative (sum z-) and positive (sum z+) responses, respectively. Vertical dashed lines indicate aridity gradient thresholds corresponding to maximal sum (z).


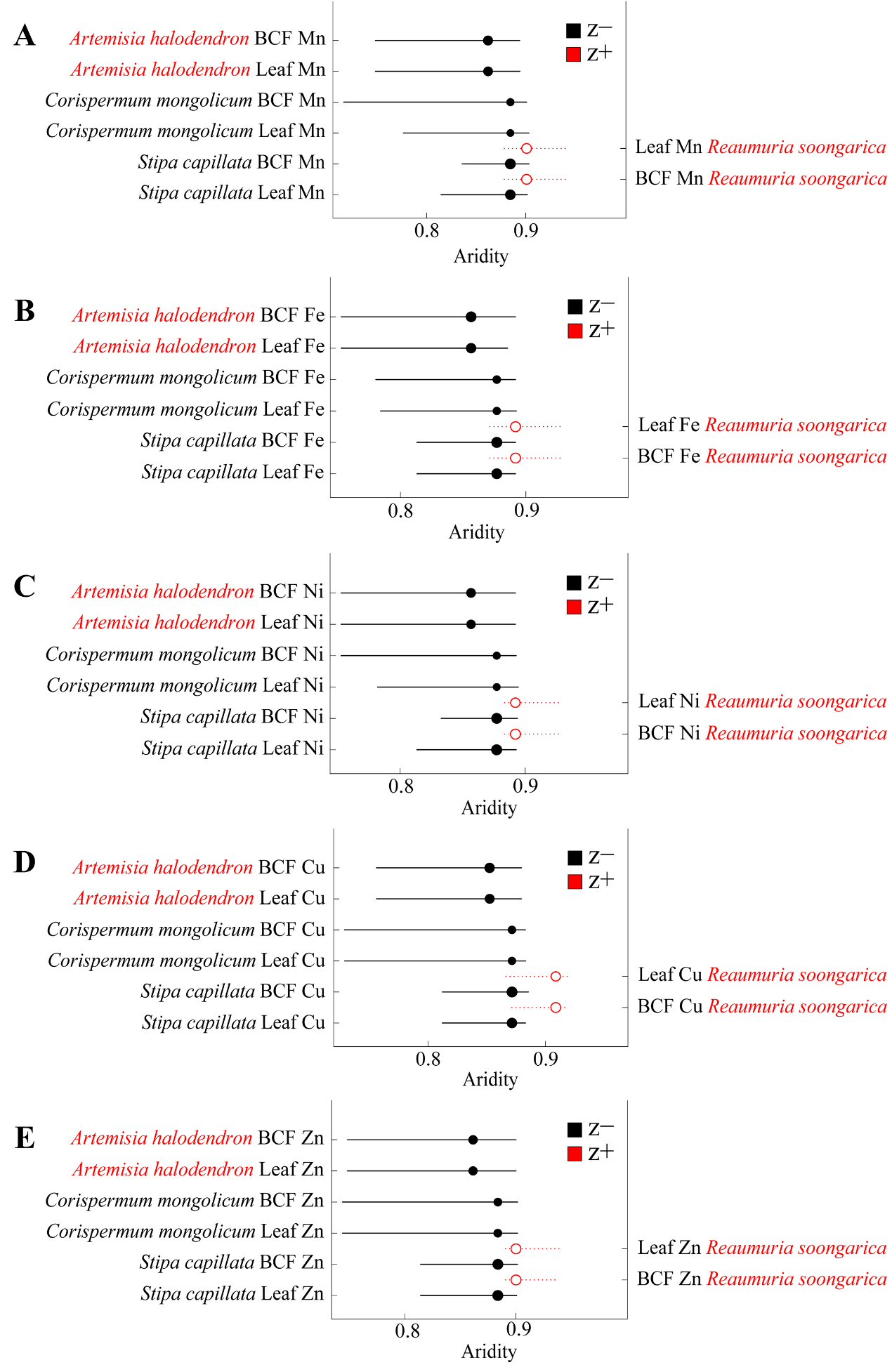


**Supplementary Figure 10 Threshold Indicator Taxa Analysis (TITAN) of leaf microelement concentrations and bioconcentration factors (BCFs) in response to aridity.** (A) Mn and the BCF of Mn, (B) Fe and the BCF of Fe, (C) Ni and the BCF of Ni, (D) Cu and the BCF of Cu and (E) Zn and the BCF of Zn. Black and red symbols denote accumulative negative (sum z-) and positive (sum z+) responses, respectively. Symbol size is in proportion to z scores (magnitude of response). Horizontal lines represent the 95% bootstrap confidence intervals. Woody and herbaceous species are shown in red and in black fonts, respectively.
